# State anxiety and depression as factors modulating and influencing postoperative pain in dental implant surgery. A prospective clinical survey

**DOI:** 10.4317/medoral.19685

**Published:** 2014-06-01

**Authors:** Rafael Gómez-de Diego, Antonio Cutando-Soriano, Javier Montero-Martín, Juan C. Prados-Frutos, Antonio López-Valverde

**Affiliations:** 1 Profesor Asociado. Departamento Ciencias de la Salud. Universidad Alfonso X El Sabio. Madrid; 2 Profesor Titular. Departamento de Estomatología. Universidad de Granada; 3 Profesor Contratado Doctor. Departamento de Cirugía. Universidad de Salamanca; 4 Profesor Titular. Departamento de Estomatología. Universidad Rey Juan Carlos. Madrid; 5 Profesor Contratado Doctor. Departamento de Cirugía. Universidad de Salamanca

## Abstract

Objetives: To determine whether preoperative state anxiety and depression modulate or influence objective and subjective postoperative pain following dental implant insertion. Study Design: Prospective, clinical study with 7-day follow-up of a sample of 105 subjects who preoperatively completed the state anxiety questionnaire (STAI-E) and Beck Depression Inventory (BDI) and postoperatively, at 2 and 7 days, recorded objective pain with the Semmes-Weinstein mechanical esthesiometer (SW test) and subjective pain with the Visual Analog Scale (VAS). Results: 85.6% and 81.5% of patients, respectively, recorded no signs of state anxiety or depression. The correlation between anxiety and depression for both maxillary bones was the lower (P=0.02). The correlation between subjective and objective pain at 2 and 7 days, and the anatomic regions intervened, was statistically significant in the mandible at day 7 (P<0.01), and highly significant (P<0.001) for the other variables. The correlation between state anxiety and objective pain at day 7 was nearly statistically significant (P=0.07). Conclusions: The correlation between state anxiety and depression, and objective and subjective pain at day 7 was not statistically significant. A strong correlation was found between objective and subjective pain in the immediate postoperative period.

** Key words:**Anxiety, depression, postoperative pain, dental implants.

## Introduction

Historically, pain has been considered a mode of somatic sensation. Today, the International Association for the Study of Pain (IASP) defines it as: “An unpleasant sensory and emotional experience associated with actual or potential tissue damage, or described in terms of such damage”, (1) leading us to understand pain as a perception implying four essential issues: sensitivity to certain tissue alterations interpreted as harmful to the integrity of the subject, the chance that pain might have a non-somatic cause justifying it and the existence of an subjective emotional component (2).

Superficial or deep somatic pain relates the damaged area to the perception of damage suffered. The surgical lesion caused by dental implant insertion triggers biochemical processes at the implant site and in the central and peripheral nervous system. This causes physiological reactions (transduction, transmission, modulation and perception) which, as we have seen, imply sensorial, motor, neurovegetative, emotional and memory responses. Consequently, the perception of pain is not just an impulse running through a nerve but an integrated process including the perception of a potentially noxious sensorial input, rapid evaluation of the noxious stimulus, the elaboration of a biological response, and the construction of an attitude towards the pain.

Dental implant treatments are a predictable therapeutic alternative widely used in dentistry to attain all three facets of oral rehabilitation: the morphological, aesthetic and functional (3-5).

Given that the characteristics of pain are modulated by physiological mechanisms like anxiety and depression, (2,6) among other factors, the present study analyzes the association between these mechanisms and objective and subjective postoperative pain. In a longitudinal prospective study, we analyze objective and subjective pain in the postoperative period following dental implant insertion as dependent variables, and correlate these with state anxiety and depression. At the outset of our study, we consider that state anxiety and depression modulate or influence objective and subjective postoperative pain.

## Material and Methods

The present study was conducted in accordance with the principles of the World Medical Association Declaration of Helsinki (8th revision, 2008 http://www.wma.net/e/polic/b3.htm) on medical research involving human subjects. All participants were made aware of the methodology and all gave written informed consent.

The study was conducted in four dental clinics in the autonomous region of Madrid (Spain) between May and December 2009. The study population consisted of 105 patients who met the inclusion criteria: age over 18 years, with no significant systemic diseases (ASA I or II), of both sexes, and indicated for dental implants. The patients were selected in consecutive non-probabilistic sampling, with the unit of analysis being each individual.

To determine objective pain we used the Semmes-Weinstein mechanical esthesiometer (SW test) (Touch-TestTM; North Coast Medical, Morgan Hill, USA). The SW test provides a numeric rating of the tactile pain threshold using a mechanical stimulus measured in grams (7). Twenty nylon monofilaments, of equal length but different diameters, provide a logarithmic scale of applied real strength and a linear scale of perceived intensity. The values obtained ( Table 1) range from 1.65 mm to 6.65 mm diameter and are equivalent to 0.008 grams and 300 grams force pressure. The filaments, starting with the smallest diameter, are applied perpendicular to the masticatory mucosa in the intervention zone until they bend. In the absence of any response, and after a 30-second pause, the test is repeated using a monofilament with a larger diameter, until the participant recognizes the stimulus.

**Table 1:**
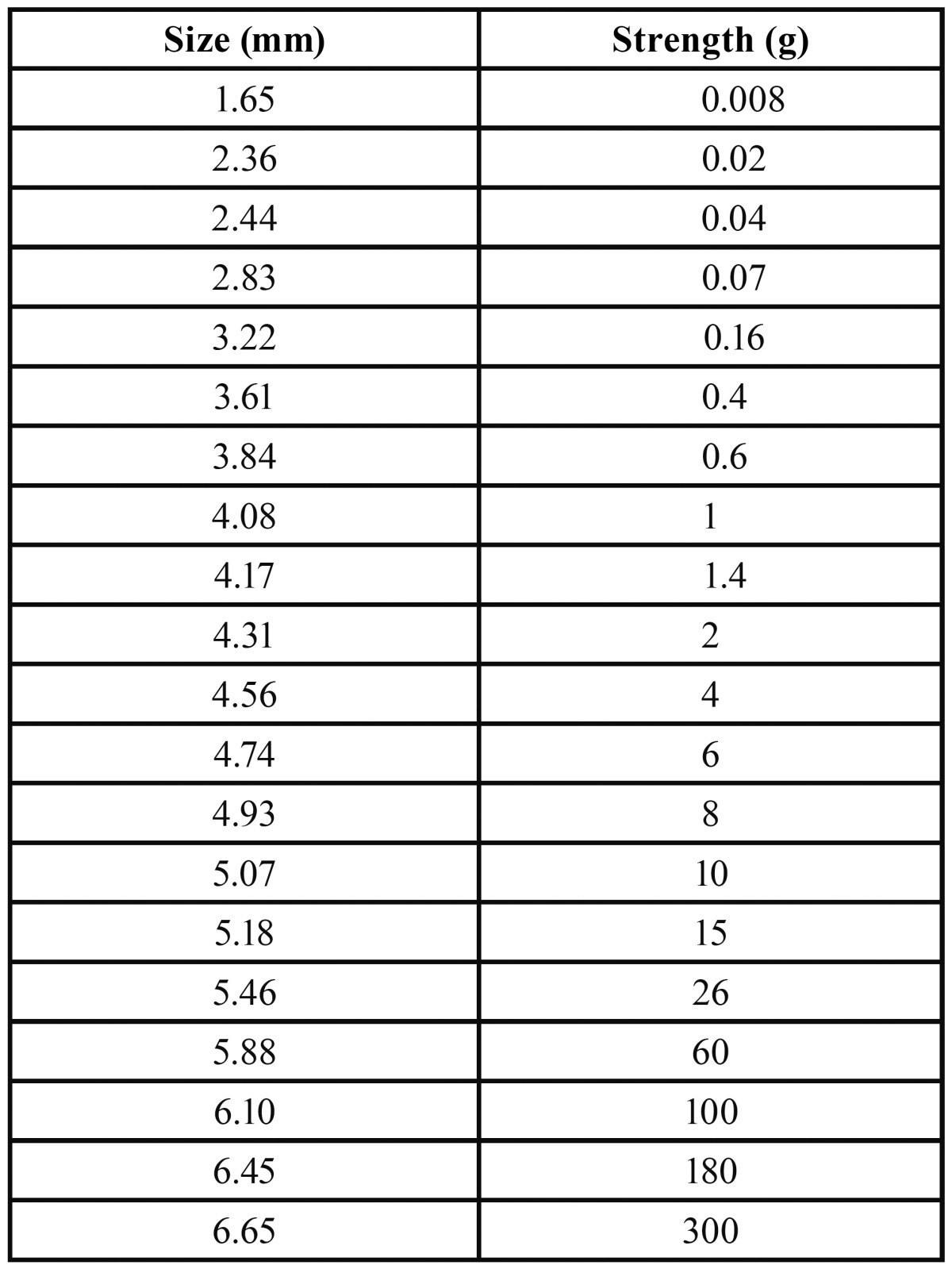
Relationship between filament diameter and strength expressed in grams.

The Visual Analog Scale (VAS) measures one-dimensional subjective pain intensity on a 10-level scale representing a continuous spectrum of the experience of pain from “0”=no pain to “10”=severe/unbearable pain (8). Patients’ perceptions of pain level were recorded.

Analysis of the variables anxiety and depression were recorded through two self-rating tests: the STAI-E questionnaire, with 20 items of 4 options each on a standard Likert scale coded from 0 to 3, establishing patient state anxiety at 19 points in men and 21 points in women (9); and the psychometric BDI inventory, with 21 multiple choice questions. Patient selected responses that best suited their current situation to determine the intensity/severity of depression. The total BDI score was the sum of response values, which vary on a 4-point scale. The range was from 0 to 63 points, with accepted cutoffs at 0 to 9 indicating no depression; 10 to 18, mild to moderate depression; 19 to 29, moderate-severe depression; and 30 points or more, severe depression (10).

The study was conducted using the standard protocol for dental implant insertion. Demographic and clinical data were collected from medical records. The independent variables–anxiety and depression–were measured the day before surgery and the dependent variables–objective and subjective pain–at 2 and 7 days following surgery.

In a descriptive analysis, we used the mean, 95% confidence interval (CI 95%) and standard deviation (SD) to check for possible errors. To analyze relation strength, we performed bivariate correlation analysis between the quantitative variables using Pear-son’s correlation coefficient and the level of statistical significance. Analysis of the nature of the relation was with multiple stepwise linear regression to quantify the effect of the independent variables that best predict the objective and subjective pain scores at day 7. In all cases, we established *P*<0.05 as statistically significant. Analysis was with SPSS 18.0 for Windows.

## Results

Of 105 patients enrolled, 8 were excluded for not presenting the necessary documentation. Data analysis ( Table 2) describes the 97 patients who participated in the study; 52.6% were women and 47.4% men, with an age range of 20 to 83 years (49.4 ± 15.4). Single implants (68.0%) and multiple implants (32.0%) were used; most (51.5%) were inserted in the mandible. Whether in the mandible or upper maxillary bone, most implants (80.5%) were in posterior anatomic regions.

**Table 2:**
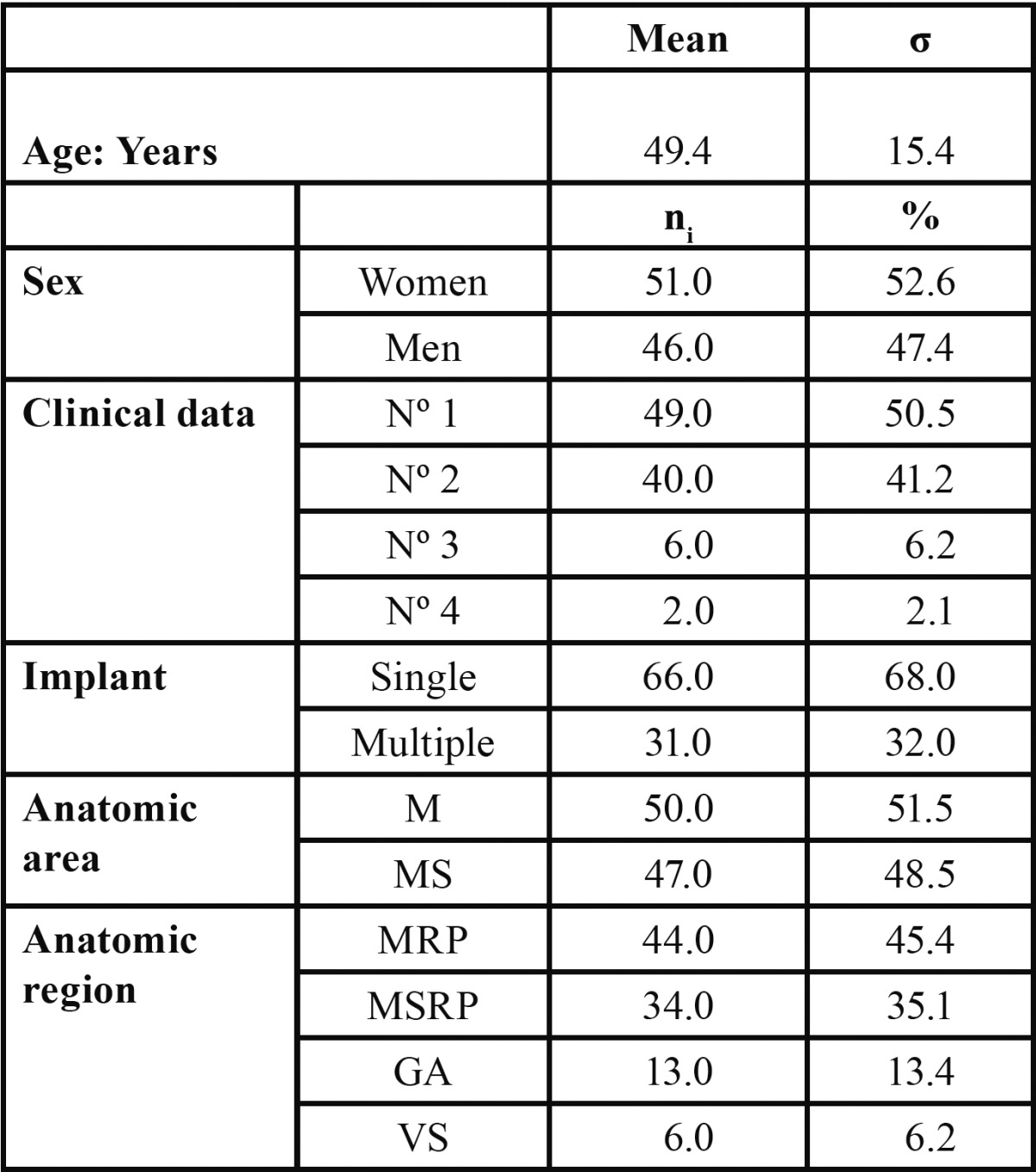
Clinical and sociodemographic description of sample (n=97).

The STAI-E questionnaire found 83 patients (85.6%) had no state anxiety and 15 (14.4%) had scores in excess of 20, reflecting a mean questionnaire total of 13.3 ± 6.8. The BDI inventory found 79 patients had no depression; 18, mild depression; and 0, moderate or severe depression; the mean total inventory score was 5.0 ± 5.4.

**Figure 1:**
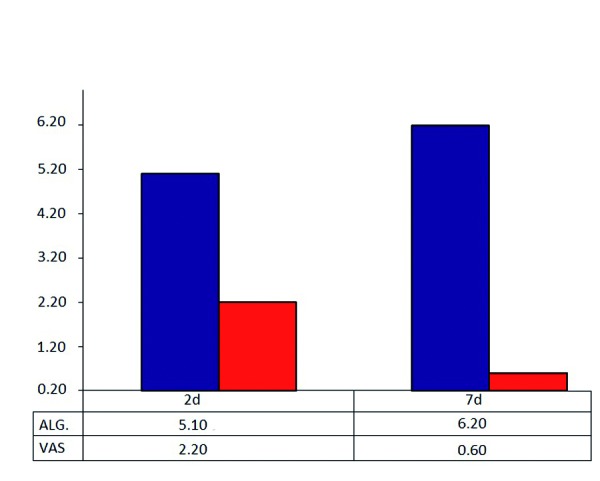
Record of objetive and subjetive painal day 2 and day 7.

Postoperative pain was determined at 2 and 7 days (Fig. 1). At day 7, the VAS found 88.6% of patients had improved, versus 11.3% who indicated feeling worse or the same; the SW test found 90.7% of patients showed improvement versus 9.3% who indicated feeling worse or the same.

Using Pearson’s correlation coefficient and the level of statistical significance for the sample, bivariate correlation analysis ( Table 3) of the relations between anxiety/depression, objective and subjective pain and the anatomic region, found a significant relation between anxiety and depression and the mandible (*P*=0.02) and a nonsignificant relation with the upper maxillary bone (*P*=0.34). The correlation between objective and subjective pain at 2 and 7 days with the same independent variable was bilateral and significant, with a greater degree of significance (*P*<0.001) in the relation between objective pain in both upper and lower maxillary bones.

**Table 3:**
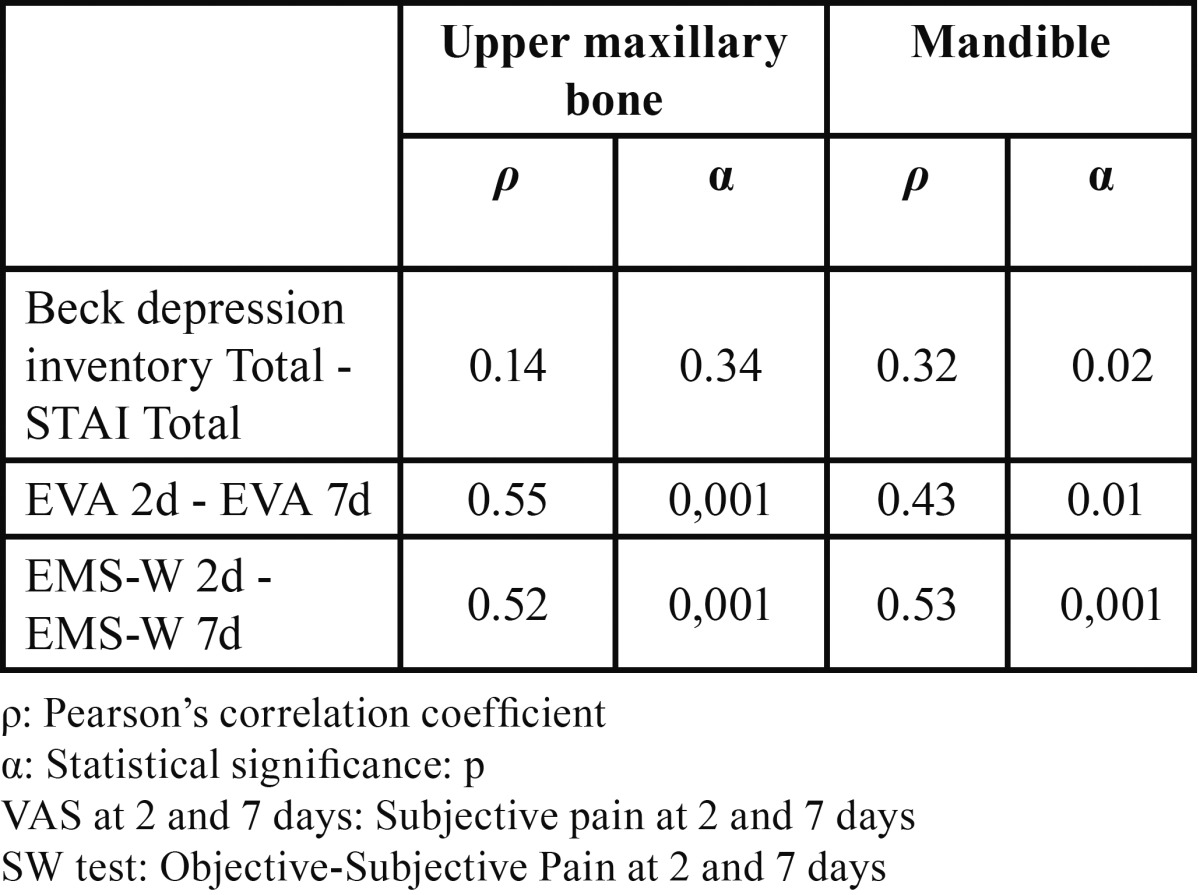
Analysis of bivariate correlations (n=97).

The correlation between anxiety and depression and objective and subjective pain at 2 and 7 days indicated the potential value of conducting multiple stepwise linear regression analysis to quantify the variables’ effect on objective and subjective pain. The general linear regression model for subjective pain at day 7 ( Table 4) found a weak predictability coefficient (R2Adj=0.24; F=9.73; gl=3, *P*<0.001), with subjective pain at day 2 being the most significant variable *P*<0.001 and that which most influenced the dependent variable (?=0.42). This led us to study the correlation between this, postoperative objective and subjective pain, and anatomic region ( Table 5). The model found a strong predictability coefficient for both anatomic regions, with objective pain at day 7, in the mandible, being the most significant (P<0.001) and most influential (?= -0.84) variable.

**Table 4:**
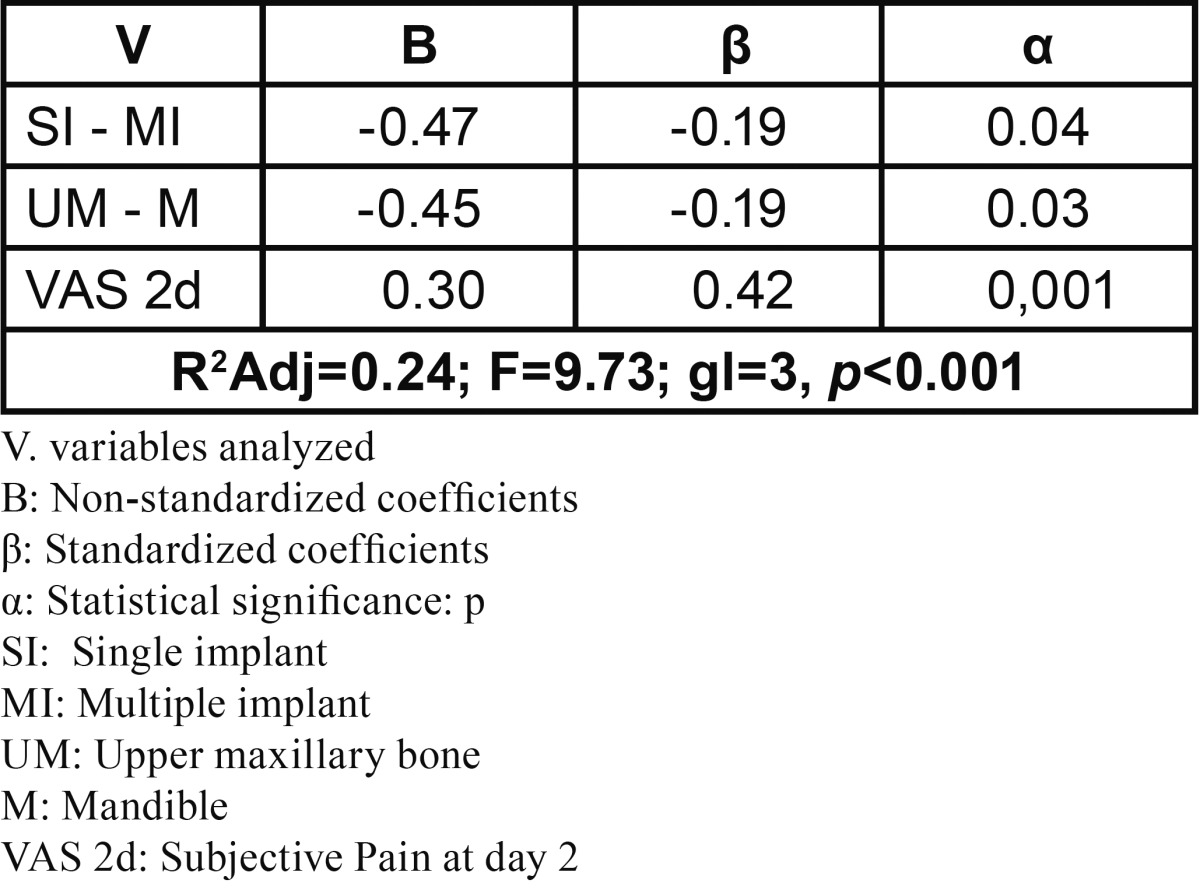
General stepwise linear regression model of subjective pain assessment at day 7 (n=97).

**Table 5:**
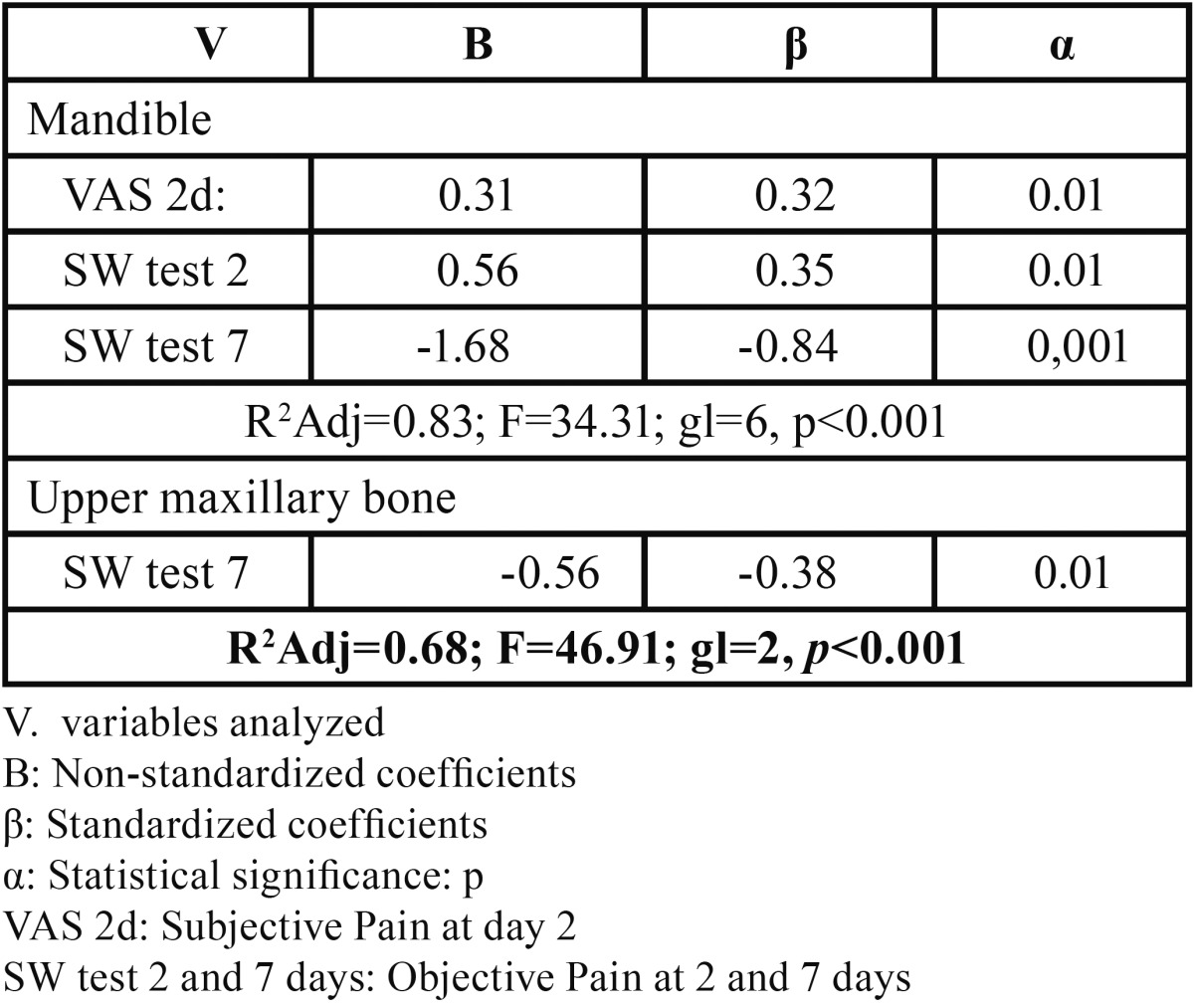
General stepwise linear regression model of subjective pain assessment at day 7 in mandible and upper maxillary bone (n=97).

The general linear regression model for objective pain at day 7 ( Table 6), recorded a moderate predictability coefficient, with postoperative objective pain at day 2 showing greater statistical significance (*P*<0.001) and state anxiety giving a value close to significant. The moderate predictive capability of the model led us to explore the correlation between the same variable and subjective pain at 2 and 7 days in relation with anatomic regions ( Table 7), with subjective pain at day 7 being the variable that recorded the greatest significance in both maxillary bones (*P*<0.001), with greater statistical weight for the mandible (?= -0.76).

**Table 6:**
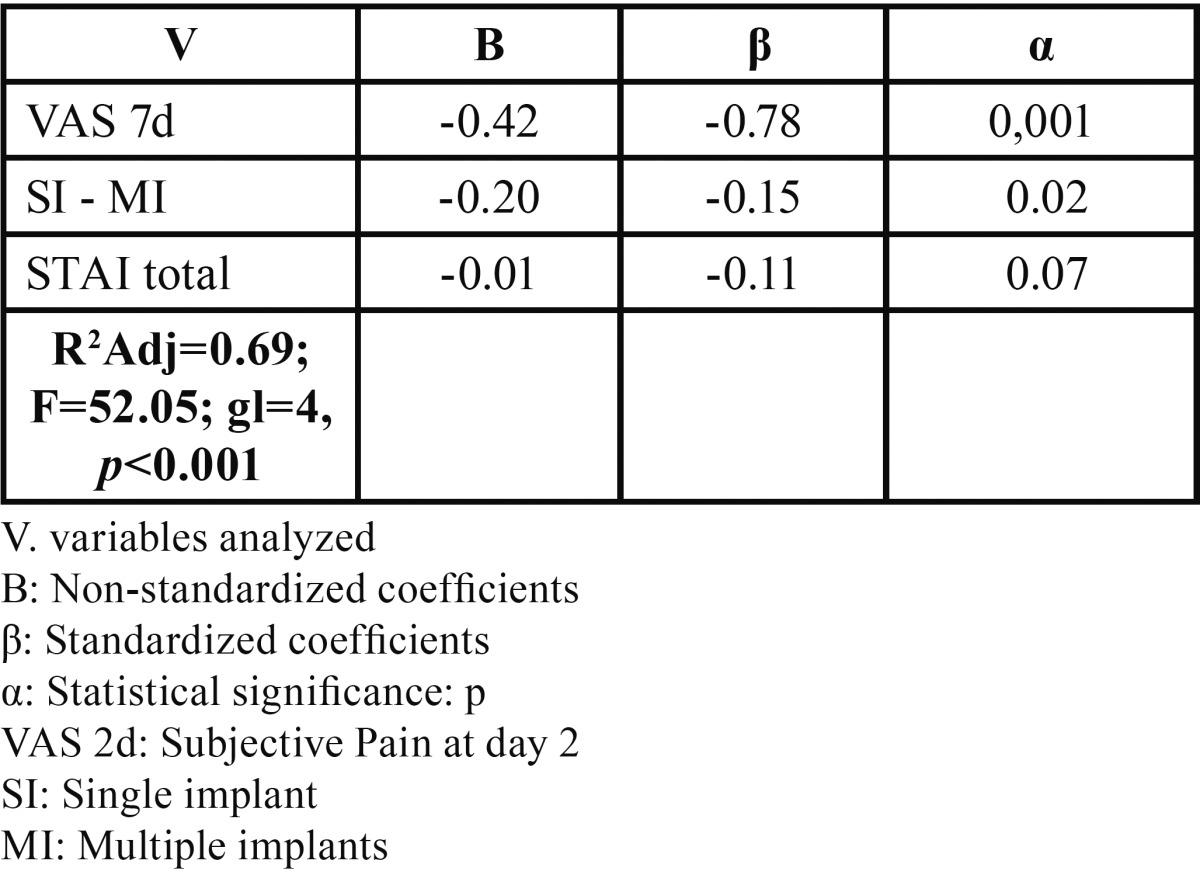
General stepwise linear regression model of objective pain assessment at day 7 (n=97).

**Table 7:**
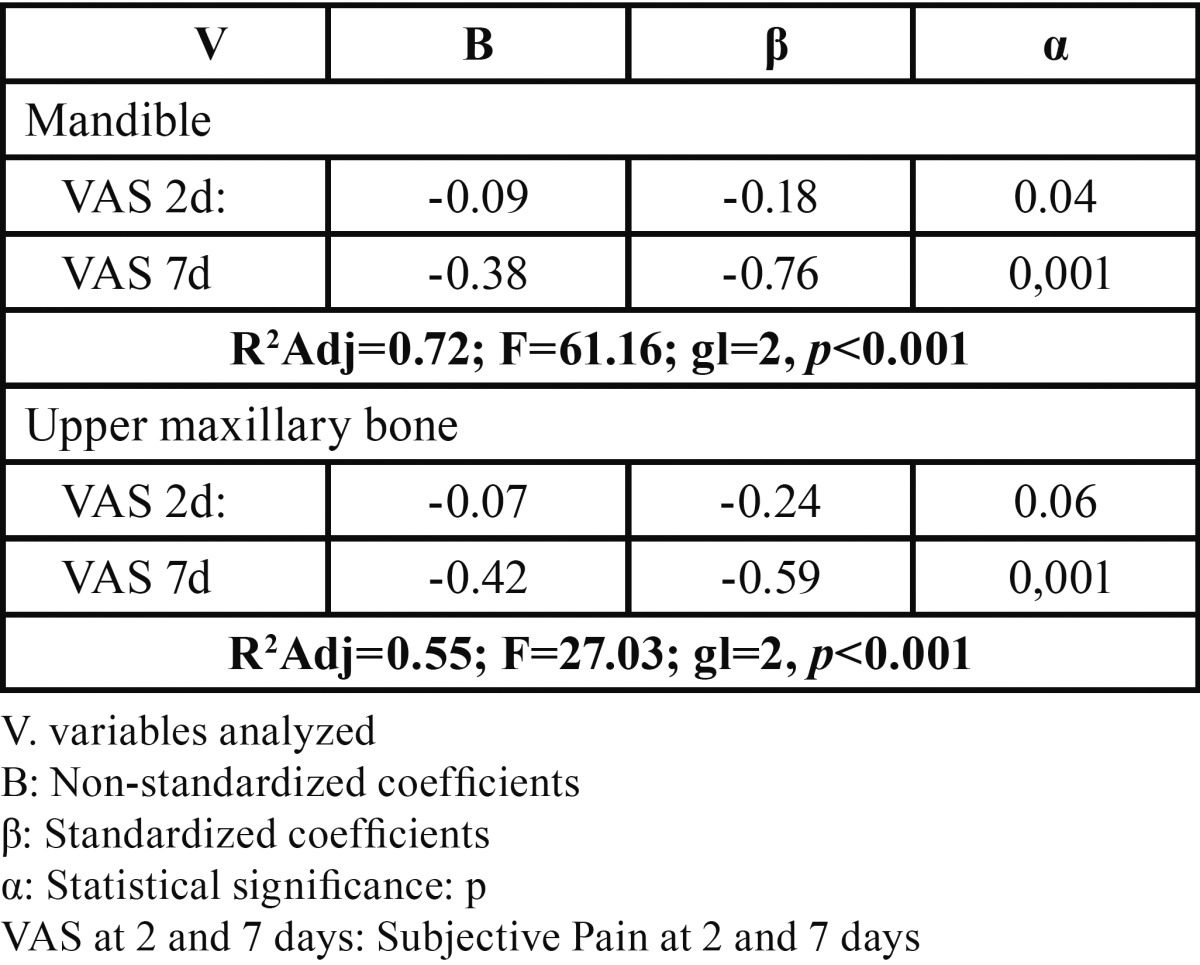
General stepwise linear regression model of objective pain assessment at day 7 in mandible and upper maxillary bone (n=97).

## Discussion

Our objective was to analyze in a prospective clinical study, objective and subjective pain in the short-term postoperative period and the correlation with state anxiety and depression. The sampling points were convenient and consecutive, so the representativity of the sample could be similar to that found in a probabilistic sample. Short-term follow-up enabled us to determine intrasubject variations and minimize information bias. The results were analyzed using descriptive analysis, bivariate correlation, and multiple stepwise linear regression to check for confounding variables.

We have applied accepted, validated instruments that have produced sound results in many epidemiological studies (2,7,10,11-15). The questionnaire used determines only the transitory “state” of anxiety when faced with a stressful situation, and not trait anxiety, as this is a stable characteristic in the individual and favours conscious nonperception of changes in apprehension and tension associated with stressful situations like dental implant surgery (2,6,9).

The age range of patients (20-83 years) and postoperative follow-up coincided with other studies of the impact of oral surgery-derived pain. González-Santana *et al.* (11) report on subjective pain at 2,4,6,12 and 24 hours in a sample of Spanish patients (range: 25-69 years); in a Canadian population (range: 35-63 years), Morin *et al.* (12) analyze postoperative pain three times a day during the first 24 hours; and in a sample of Danish patients (range: 23-77 years), Urban & Wenzel (13) conducted a 3-day follow-up of subjective pain.

Bivariate correlation analysis ( Table 3) using Pearson’s correlation coefficient found a significant correlation (*P*=0.02) between anxiety/depression and the mandible, similar to results reported by Vickers *et al.* (6) who, in their study of a sample of 438 patients, reported a statistically significant relation (*P*<0.01) between the psychological mechanisms and greater postoperative pain intensity. These same authors found a significant correlation between objective-subjective pain at 2 and 7 days and anatomic region, corroborating the validity of the materials and methods used as they coincide with the recommendations of several authors. In a sample of 65 patients receiving dental implants in the lower maxillary bone, Abarca *et al.* (14) found a statistically significant relation (*P*<0.03) when objectively determining postoperative pain using the SW test. Poort *et al.* (15) recommended using the VAS together with the SW test in their review of methods to determine sensitivity in the third branch of the trigeminal nerve in oral surgery and dental implants.

Furthermore, linear regression found no statistical significance (*P*=0.03) in the relation between anatomic region and subjective pain at day 7 ( Table 4), coinciding with Muller & Ríos Calvo (16), who studied a similar sample (n=75) with 2-year follow-up. These authors concluded the relation between postoperative pain and the anatomic region implanted was not statistically significant (*P*>0.01). Recent studies describe how painful afference of the 2nd and 3rd branch of the trigeminal nerve activates the pain protection systems such that regions of the brain affected cannot identify whether the input comes from the upper maxillary bone or mandible (17,18).

The general regression models for objective and subjective pain found statistically significant relations between subjective pain at day 2 and objective pain at day 7 (*P*<0.001) ( Table 4) and between objective and subjective pain at day 7 (*P*<0.01) ( Table 6). This indicates the relation between pain types, showing that patients with higher subjective pain scores at postoperative day 2, also present higher scores at day 7, above all in the upper maxillary bone. These results coincide with several studies 11,14: in a sample of 92 patients, Urban & Wenzel (13) found a statistically significant relation between subjective postoperative pain at day 1 and day 3, concluding that patients indicating higher levels of pain in the first test also do so in the second one. In a large sample (n=234) studied to determine subjective pain intensity at days 2 and 7, Al-Khabbaz *et al.* (19) found the level of pain at day 7 associated with that recorded on day 2 (OR=38.69).

Linear regressio models for subjective ( Table 5) and objective pain ( Table 7) at day 7 and correlated with anatomic regions, found a statistically significant relation between subjective pain and postoperative subjective pain at day 2, and objective pain at days 2 and 7 in the mandible, and with objective pain only in the upper maxillary bone ( Table 5). The objective pain model ( Table 7) found a significant relation between subjective pain at day 7 in both regions (*P*<0.001). Similar results to those reported by other authors (14,15) who propose using objective and subjective tests together to calculate the prevalence of pain. In a 12-month prospective study, Walton (20) used subjective methods and the S-W mechanical esthesiometer to analyze neurosensorial abnormalities in patients undergoing mandible implants, finding that 24% had abnormal nervous sensations–short-term dysesthesia or paresthesia. Feldman *et al.* (21) assessed subjective pain intensity in a randomized study (n=120) by comparing subperiosteal and endosseous orthodontic implant insertion with upper premolar extraction, associating pain with three surgical techniques and concluding that the group undergoing concomitant exodontia recorded greater pain at day 7 (*P*<0.01).

The general model of objective pain ( Table 6) found a nearly significant relation with state anxiety (*P*=0.07). These results are comparable with a recent study at the University of Amsterdam (Holland) (22). In 160 patients undergoing oral surgery for third molar removal, the authors concludes that anxiety does not predict surgery-related pain. This contrast with results reported by Eli *et al.* (2) who, on determining the correlation between pre- and postoperative anxiety and acute postoperative pain in an implant insertion procedure for a sample of 60 patients, found the sensation of pain associated significantly with the level of patient anxiety. Such discrepancies between authors indicate the need for more analytical case studies and randomized, controlled, double blind studies, in which patients complete both STAI state and STAI trait questionnaires.

## Conclusions

Patients more frequently reported less postoperative pain at day 7. Bivariate correlation analysis found a statistically significant relation (*P*=0.02) between anxiety and depression in the mandible. Multiple stepwise linear regression suggests that preoperative state anxiety and depression do not modulate objective and subjective postoperative pain at day 7. In the postoperative period immediately following dental implant insertion, a strong correlation exists between subjective and objective pain.
